# The Development of Empirically Derived Australian Low-Risk Gambling Limits

**DOI:** 10.3390/jcm10020167

**Published:** 2021-01-06

**Authors:** Nicki A. Dowling, George J. Youssef, Christopher Greenwood, Stephanie S. Merkouris, Aino Suomi, Robin Room

**Affiliations:** 1 School of Psychology, Deakin University, Geelong, VIC 3220, Australia; george.youssef@deakin.edu.au (G.J.Y.); christopher.greenwood@deakin.edu.au (C.G.); stephanie.merkouris@deakin.edu.au (S.S.M.); 2 Melbourne Graduate School of Education, University of Melbourne, Parkville, VIC 3010, Australia; 3 Centre for Adolescent Health, Murdoch Children’s Research Institute, Parkville, VIC 3052, Australia; 4 Research School of Population Health, The Australian National University, Canberra, ACT 2600, Australia; aino.suomi@anu.edu.au; 5 Institute of Child Protection Studies, The Australian Catholic University, Watson, ACT 2612, Australia; 6 Centre for Alcohol Policy Research, La Trobe University, Bundoora, VIC 3086, Australia; R.Room@latrobe.edu.au; 7 Centre for Social Research on Alcohol and Drugs, Department of Public Health Sciences, Stockholm University, 106 91 Stockholm, Sweden

**Keywords:** gambling, low-risk, limits, responsible gambling, guidelines, relative risk, absolute risk, gambling harm, harm

## Abstract

This study derived a set of Australian low-risk gambling limits and explored the relative and absolute risk associated with exceeding these limits. Secondary analysis of population-representative Tasmanian and Australian Capital Territory (ACT) cross-sectional (11,597 respondents) and longitudinal studies (2027 respondents) was conducted. Balancing sensitivity and specificity, the limits were: gambling frequency of 20–30 times per year; gambling expenditure of AUD $380–$615 per year (USD $240–$388 per year); gambling expenditure comprising 0.83–1.68% of gross personal income; and two types of gambling activities per year. All limits, except number of activities, predicted subsequent harm, with limits related to gambling expenditure consistently the best-performing. Exceeding the limits generally conferred a higher degree of relative and absolute risk, with gamblers exceeding the limits being 3–20 times more likely to experience harm than those who do not, and having a 5–17% risk of experiencing harm. Only 7–12% of gamblers exceeding the limits actually experienced harm. Gambling consumption lower than the limits also conferred a considerable amount of harm. Using a relative risk method, this study derived similar limits from disparate Australian states and territories. These limits can serve as working guidelines for the consideration of researchers, clinicians, and policy makers, but need to be subject to further rigorous empirical investigation.

## 1. Introduction

Gambling disorder is characterised by persistent and recurring gambling behaviour that leads to substantial impairment and disruption to personal, family, or vocational pursuits [1]. In contrast, public health frameworks conceptualise gambling problems across a continuum of risk, ranging from no risk, where no health or social problems have developed as a result of gambling, to extreme risk, where gambling behaviour results in serious problems [2]. Accordingly, the term “problem gambling” is employed in many jurisdictions to describe all forms of gambling that result in adverse consequences for individuals, families, and communities [3]. Public health perspectives employ a whole-of-population approach to inform prevention and intervention policy by identifying determinants and subsequent harms resulting from gambling policy [2]. Gambling-related harm is functionally defined as “any initial or exacerbated adverse consequence due to an engagement with gambling that leads to a decrement to the health or wellbeing of an individual, family unit, community or population” [4]. Although severe instances of problem gambling are a low base-rate phenomena [5,6,7], the burden of harm associated with gambling problems across the continuum of risk is comparable to that of depression and alcohol use disorders [8]. Harms attributed to problem gambling include financial loss, relationship breakdown, psychological distress, decrements to health, cultural harm, reduced work or study performance, and criminal activity [4]. Because problem gambling severity and harm are closely coupled but conceptually distinct constructs, harm that occurs below the clinical threshold of gambling disorder or problem gambling is still relevant to policy related to prevention and intervention [9,10]. Consistent with public health perspectives, efforts targeted at the prevention of gambling-related harm, rather than problem gambling, may be more effective as they potentially impact a much larger segment of the population [11,12].

In alcohol research, there is an accumulation of evidence on the identification of alcohol intake levels that distinguish low-risk and high-risk behaviour [13]. These cut-offs, known as low-risk drinking limits, serve as the basis for formal quantitative guidelines employed in public health initiatives worldwide [13,14]. In contrast, current “responsible gambling” guidelines generally only comprise a set of behaviours that may reduce the likelihood of gambling-related harm, such as leaving automatic teller machine cards at home and setting money limits in advance [11,15]. The development of quantitative low-risk gambling guidelines is important, given that many gamblers attempt to reduce their gambling by setting frequency, expenditure, and time limits [16]. Offering these types of guidelines fits modern ideals of consumer society, whereby it is assumed that well-informed consumers will adapt their behaviour to advice from professional organisations [13]. From a “responsible gambling” perspective, it is equally important that gambling providers are aware of the levels of gambling consumption at which harm is likely to occur [17].

There is now some evidence that the development of empirically derived quantitative low-risk gambling limits is possible. The literature has employed almost identical statistical methodologies: risk (dose-response) curves exploring the degree to which gambling behaviours are associated with gambling-related harm; receiver operating characteristic (ROC) analyses to identify optimal low-risk gambling limits; and regression modelling to examine the associations between these limits and gambling-related harm [11,12,16,18,19,20,21,22]. Because there is no standard unit of gambling, the dose-response relationship in these studies has been explored across multiple dimensions of gambling consumption, most commonly gambling frequency, gambling expenditure, and gambling expenditure as a proportion of income. In the absence of a validated measure of gambling-related harm, gambling-related harm has been defined using diagnostic criteria or subsets of items from measures of problem gambling severity, such as the Problem Gambling Severity Index (PGSI) and the South Oaks Gambling Screen (SOGS).

### 1.1. Development of Low-Risk Gambling Limits in Population-Representative Samples

Low-risk gambling limits have been identified in cross-sectional population-representative studies in Canada [11,12,16,19] and Germany [18]. Although these limits generally yield moderate levels of classification accuracy [11,12,16,18,19] and have generally been robust to variations in definitions of harm [11,12,16,18,23], superior ROC parameters are generally produced when gambling-related harm is defined as two or more negative consequences [11,12,16,23]. These studies have identified relatively consistent limits: gambling no more than 0.6–5 times per month; spending no more than the equivalent of USD $93–$720 per year on gambling; spending no more than 1–3% of gross household income on gambling; and gambling on no more than 2–4 activities per year. A significant proportion of gamblers in the Canadian general population (11–41%) exceed at least one of these limits [11,19] and longitudinal research suggests that gambling at levels beyond these limits is indicative of future gambling-related harm [19,24].

In the absence of a conceptual rationale for maximising sensitivity or specificity, these low-risk gambling limits were identified using a relative risk approach that involved giving equal weighting to sensitivity (the ability of a low-risk gambling limit to accurately identify individuals experiencing gambling-related harm) and specificity (the ability of a low-risk gambling limit to accurately identify individuals not experiencing gambling related harm). Currie et al. [12], however, has expressed concerns that high false positive rates may diminish the credibility of low-risk limits. To date, Currie et al. [19] is the only study that has employed ROC analyses that have not balanced sensitivity and specificity. In this study of two independently conducted Canadian cohort datasets, analyses that gave equal weighting to sensitivity and specificity resulted in some poor specificity values, which increased the proportion of false positives. The authors therefore employed an alternative method in which selected cut-offs maximised specificity (while maintaining sensitivity at 0.70 or higher), resulting in higher limits. This approach contrasts with the long-standing conservative approach to the prevention and treatment of alcohol use problems, in which it is assumed that the consequences of setting thresholds so high that a substantial proportion of adverse outcomes occur at consumption levels below that threshold (low sensitivity) are more serious than the consequences of setting thresholds so low that the majority of people exceeding them do not experience any adverse consequences (low positive predictive value) [25]. This is consistent with the public health perspective’s general commitment to the “precautionary principle”, which asserts that scientific uncertainty must not be used as a reason to ignore or postpone preventive action when there are threats of serious damage [26].

Moreover, the alcohol literature has identified low-risk drinking limits based on an absolute risk approach, in which limits were statistically determined based on tolerable levels of absolute risk [13,27,28]. This approach involves setting the low-risk criterion so that the lifetime chances of dying of an alcohol-involved condition for a person who drinks consistently below the criterion is below some risk level, which is sometimes discussed as 1 in 100 [13,27,28], or even as high as 1 in 1000 [29]. Markham et al. [30] has recently argued that, like low-risk drinking limits, low-risk gambling limits should also be made on the basis of the amount of absolute risk that can be tolerated. In contrast to previous research that described gambling risk (dose-response) curves as J-shaped [11,16,19], whereby the chances of experiencing gambling-related harm remain low at low levels of gambling consumption then increase sharply when a certain threshold of gambling behaviour is reached, Markham et al. [30] employed a different methodology to estimate that the risk curves for gambling expenditure are either r-shaped or linear, suggesting that increasing expenditure increases risk of harm even at relatively low levels of consumption. While risk curves are not themselves employed to identify optimal cut-offs, they may imply that low-risk gambling limits should be made on the basis of the amount of absolute risk that can be tolerated [30].

### 1.2. Study Aims

Empirically-derived low-risk gambling limits can be used to inform the development of formal quantitative low-risk gambling guidelines that can usefully augment the behavioural responsible gambling guidelines that are currently available [11,15]. It remains unclear, however, whether the limits identified predominantly in Canadian general population samples are generalizable to other jurisdictions, such as Australia, given differences in gambling availability, regulation, and treatment provision [11,15,18]. Both countries have adopted a public health perspective [31] and have generally similar national or standardised estimates of problem gambling [5,6,7,32,33]. However, despite having only three-quarters of the population of Canada, Australia’s total gambling losses are 50% higher than Canada’s gambling losses [33]. Australia has the highest per capita gambling losses in the world, whereby gambling losses per resident adult are nearly 60% higher than in Canada [31]. Moreover, electronic gaming machines (EGMs), which generate the majority of gambling losses, account for a much higher proportion of gambling losses in Australia; and Australia has twice as many EGMs and one third the number of persons per EGM as Canada [31]. The aims of this study were therefore to replicate previous research by deriving a set of Australian low-risk gambling limits; identifying the proportion of the population exceeding these limits; and exploring whether gambling at levels beyond these limits is longitudinally associated with harm.

However, given the argument that limits might better be made on the basis of tolerable levels of risk [30] and the emphasis on predictive values in the alcohol literature [25], this study also aimed to extend the existing literature by identifying the relative and absolute risk associated with exceeding the limits; and identifying the positive predictive values (PPVs; predicted proportion of gamblers exceeding the limits who actually experience harm) and negative predictive values (NPVs; predicted proportion of gamblers remaining within the limits who do not actually experience harm) associated with the limits. A final exploratory aim was to examine the impact of maximising sensitivity and specificity on these limits and their associated predictive values.

## 2. Experimental Section

### 2.1. Participants and Procedure

This study involved the secondary analysis of population data from the second and third Social and Economic Impact Study (SEIS) of Gambling in Tasmania [34,35], the 2014 Survey on Gambling, Health, and Wellbeing in the Australian Capital Territory (ACT) [36], and the Tasmanian Longitudinal Gambling Study (TLGS) [37]. These Computer Assisted Telephone Interviewing (CATI) surveys were selected for analysis as they are among the few available population-representative studies in Australia to collect continuous expenditure data across multiple gambling activities (see Table 1 for methodological details and sample descriptive statistics for the surveys employed in this study). The data from the second and third SEIS surveys were merged, as they employed almost identical measures and few differences in gambling participation and problem gambling severity were identified over the 2.5 years between surveys [34]. In contrast, the Tasmanian and ACT datasets were analysed separately, as it is a well-known statistical phenomenon that it is inappropriate to merge independent datasets as there may be unknown or paradoxical consequences due to unmeasured differences (e.g., Simpson’s paradox) [38]. This can lead to trends appearing in different groups of data but disappearing or reversing when these groups are crudely combined. This was an important consideration given these two jurisdictions display very disparate socio-demographic and gambling participation profiles (Supplementary Table S1). Thus, each dataset was weighted for the state/territory population and each construct was operationalised slightly differently in each state/territory.

### 2.2. Measures

Indices of gambling consumption on which the low-risk gambling limits are derived were gambling frequency, gambling expenditure, gambling expenditure as a proportion of personal income, and number of types of gambling activities (Supplementary Table S2). Given evidence that excluding people who only played lottery derives almost identical low-risk limits [11,16,23], and that, most stakeholders agree that all gambling activities should be included in the development of overall low-risk limits [15,23], these gambling consumption indices were based on all gambling activities, including lotteries.

Given that no validated measures of gambling-related harm were available when the surveys were conducted, harm was measured using selected items from the nine-item Problem Gambling Severity Index [3]. Using the PGSI, respondents indicate how often each item applied to them in the last 12 months on a four-point scale: (0) never, (1) sometimes, (2) most of the time, and (3) almost always. It has been argued that, although the PGSI is a measure of problem gambling severity, it is a viable instrument to measure harm because it has a focus on the negative consequences of gambling rather than on behavioural symptoms [12]. It has been argued in the previous low-risk limits literature that the PGSI comprises a subset of 7 items measuring negative consequences of gambling and a subset of 2 items measuring behavioural symptoms of gambling [12,16]. This classification of negative consequence and behavioural symptom items is not consistent with the classification employed in the development of the PGSI [3], but was retained to allow comparisons to previous research (Supplementary Table S3). Given that superior ROC parameters are generally produced when gambling-related harm is defined as two or more negative consequences [11,12,16,23], this study explored the optimal cut-offs using endorsement of two or more gambling-related problems among the seven negative consequence items of the PGSI as the selected definition of harm. This definition of harm is based on dichotomous, presence-absence scoring of individual items, whereby respondents who endorse a negative consequence PGSI item as occurring “sometimes”, “most of the time”, or “all of the time” are coded as experiencing a gambling-related harm.

### 2.3. Data Analytic Strategy

Unless otherwise stated, all analyses were conducted in Stata-14 [39], employed weighted data from past-year gamblers, and conducted statistical testing at *p* < 0.05. All inferential analyses were performed using a robust variance estimator to adjust for heteroscedasticity. All analyses were conducted using available cases, excluding missing data. Previously conducted bootstrapped linear regression analyses suggested that the shape of the dose-response risk curves for both datasets was either linear or r-shaped [23]. Low-risk gambling limits were identified using ROC analyses across the multiple gambling indices. After plotting the sensitivity and 1-specificity for each level of gambling consumption, the area under the curve (AUC) of the resulting ROC graph was calculated. AUC values, which provide a general index of the classification performance of a test, were interpreted according to established guidelines: small (0.50–0.70), moderate (0.70–0.90), and high (>0.90) [40]; moderate to high classification accuracy was considered acceptable in this study [18,40]. The level of gambling consumption that had the maximum Youden Index value [41] relative to all other levels was deemed the optimal cut-off (with equal weighting given to sensitivity and specificity) [42]. This method equally minimises false positives and false negatives [43]. A series of binary logistic regressions that included the interaction effects between the low-risk gambling limits and sex and age in predicting gambling-related harm were calculated. The proportion of the population (including non-gamblers) and the proportion of gamblers who exceeded the limits was calculated, and for analyses concerning the population sample, all non-gamblers were classified as not meeting the limit. We also explored the proportion of gamblers exceeding multiple proposed low-risk gambling limits. A second and third set of exploratory ROC analyses were conducted in which sensitivity and specificity were maximised, while limiting each of specificity and sensitivity to no lower than 0.50.

Logistic regression analyses (controlling for socio-demographic characteristics) were employed to explore the degree to which exceeding each of the limits at Wave 1 of the TLGS predicted subsequent (binary) harm in Waves 2 and 3. These analyses were then repeated with the other limits as additional covariates. Wave 1 data was used to predict subsequent harm in both waves to determine the stability of the predictive ability of the limits over a longer period of time. The gambling expenditure as a proportion of gross personal income limit was removed due to multicollinearity with the gambling expenditure limit. These analyses were conducted with non-gamblers classified as having no harm and with weights for the complete sample who provided longitudinal data.

A set of sliding scales (using unweighted data) was created to highlight the relative risk (proportion of gamblers experiencing harm in those exceeding the limit divided by proportion of gamblers experiencing harm in those not exceeding the limit) and absolute risk (number of people experiencing harm in relation to the population at risk) associated with different levels of gambling consumption. The limits were used as a basic metric and the relative and absolute risk was calculated for gambling consumption at increasing multiples below, at, and above each of the limits.

Absolute risk was also investigated by evaluating the PPVs and NPVs for each of the limits based on the prevalence of harm. The PPVs and NPVs were explored in both the population and gambling samples from the Tasmanian SEIS and ACT datasets. PPVs and NPVs were then graphed using the sensitivity and specificity from ROC analyses against prevalence rates of harm ranging from 0% to 100% prevalence to illustrate that PPVs and NPVs are influenced by the prevalence of gambling-related harm in the population that is being tested.

## 3. Results

### 3.1. Identification of Low-Risk Gambling Limits

The optimal cut-offs in ROC analyses using the Youden Index were explored across the multiple gambling indices and gambling-related harm in the Tasmanian SEIS and ACT datasets (Table 2). The limits were relatively consistent across the two jurisdictions, although the ACT limits were consistently more conservative. The low-risk limits, which were all in the moderate classification accuracy range (AUC = 0.70–0.90), were exceeding any of the following: gambling frequency of 20–30 times per year; gambling expenditure of AUD $380–$615 per year (USD $240–$388 per year); gambling expenditure comprising 0.83–1.68% of personal gross personal income; and two types of gambling activities per year. There were no significant interaction effects between the low-risk limits and gender or age in predicting gambling-related harm.

**Figure 1:**
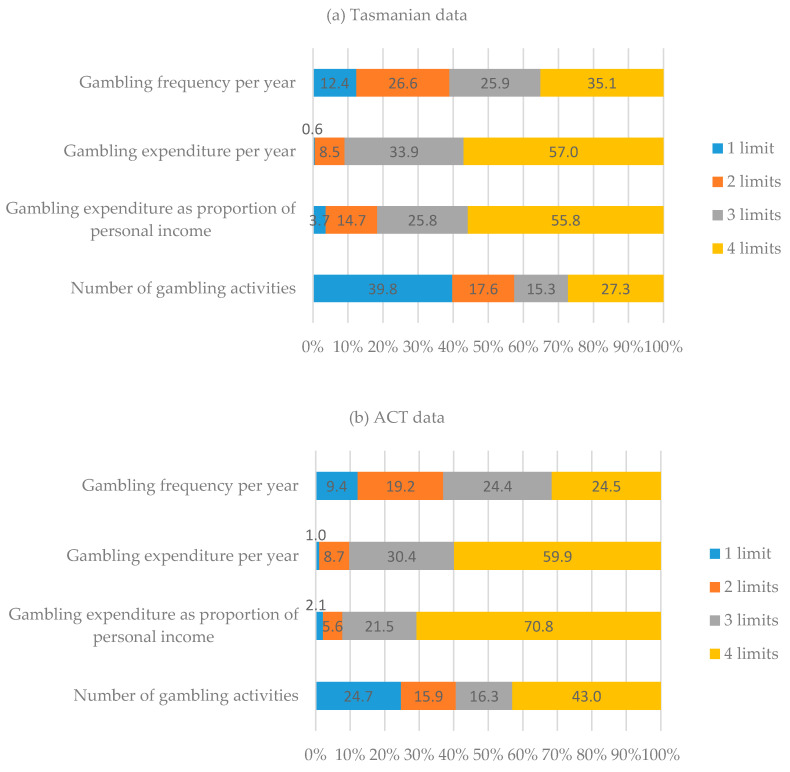
Proportion of gamblers exceeding multiple low-risk gambling limits ^a^ (**a**) in Tasmania; (**b**) in Australian Capital Territory (ACT). ^a^ Endorsement of two or more PGSI negative consequence items selected as definition for derivation of low-risk gambling limits.

Table 2 reveals that the largest proportion of both samples exceeded the number of types of gambling activities limit, followed by the gambling frequency limit. The smallest proportion of both samples exceeded the limits relating to gambling expenditure (gambling expenditure per year and gambling expenditure as a proportion of gross personal income). Of the gamblers who exceeded the limits, relatively few exceeded only one limit (0.6–39.8%) and many exceeded all four limits (24.5–70.8%). This is particularly true for the limits relating to gambling expenditure and gambling expenditure as a proportion of gross personal income (Figure 1).

### 3.2. Longitudinal Evaluation of the Low-Risk Gambling Limits

After controlling for socio-demographic characteristics, exceeding the gambling frequency (OR = 6.11–14.26) and gambling expenditure as a proportion of gross personal income (OR = 15.39–21.11) limits in Wave 1 of the TLGS significantly predicted gambling-related harm in both Waves 2 and 3; and exceeding the gambling expenditure limit in Wave 1 significantly predicted harm in Wave 2 only (OR = 14.23) (Table 3). After controlling for the other limits and socio-demographic characteristics, exceeding the gambling expenditure limit in Wave 1 significantly independently predicted harm in Wave 2 (OR = 10.67); and exceeding the gambling frequency limit in Wave 1 significantly independently predicted harm in Wave 3 (OR = 20.45) (Table 3).

### 3.3. The Relative and Absolute Risk Associated with Exceeding the Low-Risk Gambling Limits

The relative and absolute risk calculations in the sliding scales (Table 4) indicate that exceeding the limits was associated with a high degree of risk for gambling-related harm relative to gamblers who did not exceed the limits, particularly in relation to both the gambling expenditure and gambling expenditure as a proportion of gross personal income limits. In relative risk terms, gamblers exceeding the limits were 3.4 to 20.2 times more likely than gamblers who did not exceed the limits to report gambling-related harm. In absolute risk terms, gamblers exceeding the limits had a 4.7 to 17.1% risk of experiencing harm. Risk ratios only slightly increased as gambling consumption increased, while the degree of absolute risk incrementally increased as gambling consumption increased. Levels of gambling consumption that are lower than the low-risk limits also conferred a considerable degree of risk.

### 3.4. Positive and Negative Predictive Values Associated with the Low-Risk Gambling Limits

In the Tasmanian SEIS and ACT surveys, between 6.8% and 11.5% of gamblers (3.7–7.4% of population) who exceeded the limits would actually be experiencing gambling-related harm (PPVs); and between 98.3% and 98.9% of gamblers (99.0–99.4% of population) who stayed within the limits would not be experiencing harm (NPVs) (Table 5). The highest PPVs and NPVs were displayed by the limits relating to gambling expenditure (gambling expenditure and gambling expenditure as a proportion of gross personal income). The PPVs and NPVs using the sensitivity and specificity from ROC analyses against prevalence rates of harm ranging from 0% to 100% prevalence (Supplementary Figure S1). This figure reveals the predictive power of the limits in different samples with different base-rate prevalence of harm, whereby PPVs are much higher in settings in which there is a high base-rate prevalence of harm and PPVs are much lower in settings in which there is a low base-rate prevalence of harm.

### 3.5. Effect of Maximising Sensitivity and Specificity on the Low-Risk Gambling Limits

The cut-offs in ROC analyses were identified after maximising sensitivity and maximising specificity (Table 6). After maximising sensitivity, the limits, which were all in the moderate classification accuracy range, were exceeding any of the following: a gambling frequency of 10 to 16 times per year; a gambling expenditure of AUD $115 to $169 per year (USD $72 to $106 per year); a gambling expenditure comprising 0.24 to 0.51% of an individual’s gross personal income; and 2 types of gambling activities per year (Table 6). Between 6.2% and 6.9% of gamblers (3.4–4.4% of population) who exceeded these limits would actually be experiencing gambling-related harm (PPVs); and between 98.3% and 99.5% of gamblers (99.1–99.7% of population) who stayed within these limits would not be experiencing gambling-related harm (NPVs) (Table 7).

After maximising specificity, the limits, which were all in the moderate classification accuracy range, were exceeding any of the following: a gambling frequency of 49 to 65 times per year; a gambling expenditure of AUD $1380 to $2306 per year (USD $870 to $1455); a gambling expenditure comprising 3.03 to 6.19% of an individual’s gross personal income; and two to three types of gambling activities per year (Table 6). Between 6.8% and 21.4% of gamblers (3.7–14.4% of population) who exceeded these limits would actually be experiencing gambling-related harm (PPVs); and between 97.8% and 98.3% of gamblers (98.6–99.1% of population) who stayed within these limits would not be experiencing gambling-related harm (NPVs) (Table 7).

## 4. Discussion

### 4.1. Identification of Australian Low-Risk Gambling Limits

This study aimed to replicate previous research predominantly conducted in Canada by identifying a set of empirically based low-risk gambling limits that can be used to inform the development of low-risk gambling guidelines for Australia. The definition of harm based on two or more of the seven negative consequence PGSI items produced good ROC parameters in the current study and in previous research [11,12,16,23], suggesting that people endorsing harms in two different areas can be reasonably viewed as experiencing gambling-related harm [11]. In the current study, this definition also captured a relatively high proportion of the population (1.9–2.3%), although these estimates are lower than those identified in Canadian samples (4.2–6.0%) [11,16]. The limits identified in these Australian samples are generally at the lower end of the range identified in previous Canadian studies [11,12,16,19]. In this study, similar low-risk gambling limits were identified across jurisdictions, although the ACT limits were consistently slightly more conservative than the Tasmanian limits. This is not unexpected given the differences in the socio-demographic and gambling participation profiles between these jurisdictions [34,35,36,44].

Consistent with previous longitudinal research [11,19], the limits generally predicted subsequent gambling-related harm (with the exception of number of types of gambling activities). The limits relating to gambling frequency and gambling expenditure (gambling expenditure and gambling expenditure as a proportion of gross personal income) significantly longitudinally predicted gambling-related harm in at least one wave. However, only exceeding the gambling expenditure limit (from Wave 1 to Wave 2 only) and the gambling frequency limit (from Wave 1 to Wave 3 only) independently predicted subsequent gambling-related harm after controlling for the other proposed low-risk gambling limits.

There were no significant interaction effects of either gender or age with the low-risk gambling limits to predict gambling-related harm. These findings suggest that each limit predicts gambling-related harm equally for men and women and across age categories. This is consistent with previous literature that suggests that the dose-response relationship between gambling behaviour and gambling-related harm is similar for men and women [11,16,19] and across age groups [19]. Moreover, few experts and members of the public agree that separate low-risk gambling guidelines should be available for men and women [23]. Taken together, these findings suggest that the calculation of gender- and age-specific limits is unnecessary.

Gamblers exceeding the limits are the target audience for the promotion of limits. In this study, this group (26–60%) was considerably higher than in Canadian samples [11,19], as might be expected given that gambling revenue per adult is over twice as much in Australia [31]. Interestingly, the findings indicate that a considerable proportion of gamblers exceeding a particular limit also exceeded other limits, suggesting that the promotion of even one limit will also likely identify gamblers who exceed other limits. The gambling expenditure limits were consistently the best-performing, but gambling expenditure as a proportion of income may be preferable as it is not confounded by annual income and provides a standardised index across the gambling population [19,21,45,46]. Although this limit requires gamblers to calculate an individual expenditure limit, a chart highlighting suggested expenditure based on gross personal income could enhance dissemination of this limit.

### 4.2. The Relative and Absolute Risk Associated with Exceeding the Low-Risk Gambling Limits

This study aimed to extend the available literature by identifying the relative and absolute risk associated with exceeding the limits. The relative risk calculations indicated that exceeding the limits generally confers a higher degree of risk for gambling-related harm. The absolute risk calculations revealed that the degree of absolute risk incrementally increased as gambling consumption increased. However, only a relatively small proportion of gamblers exceeding the limits (7–12%) will actually be experiencing harm. These low PPVs, which are not unexpected given the relatively low base-rate prevalence of gambling-related harm in the population, are not unique to gambling. Similarly, low PPVs have been identified for low-risk drinking limits, which are consistently less than 10% and usually less than 5%, regardless of which risk drinking measure is employed [25]. They may be explained by the presence of factors that could result in gamblers experiencing harm independent of consumption level or the failure of the PGSI to capture all possible gambling-related harms. These limits will, however, identify a much higher proportion of people exceeding the limits who are actually experiencing gambling-related harm in higher-prevalence settings. Settings in which there is likely a high proportion of people experiencing gambling-related harm, such as gambling venues, mental health services, alcohol and drug use services, general practitioner (GP) offices, and gambling counselling services are therefore appropriate settings in which to promote low-risk gambling limits.

Gambling consumption lower than the low-risk gambling limits identified in this study also conferred a considerable degree of risk. Combined with the previously identified linear or r-shaped risk curves identified using these datasets [23], these findings raise questions regarding the degree to which there is any level of gambling behaviour that is not associated with harm [30]. This may indicate that the low-risk limits are set too high or that a low-risk guideline such as that employed for nicotine is required, in which the population is advised that the lowest-risk choice is not to gamble for money at all, but staying below all of these guidelines will keep the risk of harm relatively low if they do gamble. Alternatively, it may mean that limits should be statistically determined based on tolerable levels of absolute risk [30]. This approach was adopted in determining Australia’s 2020 low-risk drinking guidelines, in which a standard of 1 in 100 was employed as “an acceptable risk from drinking in the context of present-day Australian society” [13,27,28]. Future research ascertaining the actual shape of risk curves using different methodologies and for different indices of gambling consumption [19,30], as well as consideration of the amount of tolerable absolute risk from gambling [47], is required before deriving low-risk gambling limits using absolute risk methods.

### 4.3. Effect of Maximising Sensitivity and Specificity on Low-Risk Gambling Limits

The ROC analyses repeated with sensitivity maximised produced limits that were approximately one-third to one-half the limits identified by balancing sensitivity and specificity, with a slight increase in false positives (6–7% of identified gamblers experienced gambling-related harm). In contrast, the ROC analyses repeated with specificity maximised produced limits that were generally two to four times higher than those which balanced sensitivity and specificity. Although there was a slight reduction in false positives (7–21% of identified gamblers experienced gambling-related harm), these limits are predominantly based on less than acceptable sensitivity estimates. In the absence of a consensus regarding the maximisation of sensitivity or specificity, these levels are not recommended for adoption as the proposed low-risk limits. Such limits are intended as a guideline to the population on ways of limiting the chances of serious gambling-related harm, and in this context, “false negatives” (i.e., harm even though the guideline was followed) matter a great deal more than “false positives” (i.e., lack of harm where the guideline was exceeded) [25]. Accordingly, there is a long-standing tradition in health research that sensitivity is generally given at least as much emphasis as specificity and PPV, despite specificity contributing far more strongly to overall predictive accuracy [25].

### 4.4. Study Limitations

Limitations include deficiencies in the representativeness of the survey samples relative to the general population which, for instance, underrepresent younger adults. Although the data were weighted to compensate for this under-representation, future research is required to explore the applicability of the derived limits to young people specifically. Another deficiency is the use of self-reported measures of gambling consumption and gambling-related harm. Despite concern that self-reports underestimate gambling consumption [48], a strong argument can be made for basing low-risk limits on self-reported data because they best reflect the perceptions of gamblers when they consider the relevance of the limits to their consumption [15,16]. Consistent with the under-reporting of alcohol consumption [49], the most serious consequence of under-reporting gambling consumption is that the limits could be somewhat conservative [11,15], which is preferable if the intent is to provide the general public with guidance about safe gambling levels [11]. This tendency to under-report may support the use of a limit range or the upper threshold of a range rather than precise figures [16]. Despite the use of self-reported measurement of harm for the low-risk drinking guidelines, these harms are usually based on relatively more objective indicators of harm, such as injury from specific drinking occasions and total mortality [13,27,28]. In contrast, given the behavioural nature of gambling, the low-risk gambling limits are based on more subjective and “softer” measures of harm. Another limitation was the measurement of gambling-related harm using item subsets of the PGSI, a measure of problem gambling severity, in the absence of available validated measures of harm. Given the emergence of research in this area [50,51,52], it is recommended that future research attempting to identify low-risk limits employ a psychometrically valid measure of gambling-related harm or more objective indicators of harm. A final limitation of this study relates to the categorical measurement of personal income, in which there was a high amount of missing data and broad bandwidth intervals. There is a need for more precise measurement of income in future studies.

### 4.5. Study Implications

The identification of low-risk gambling limits can support efforts targeted at the prevention of gambling-related harm, rather than problem gambling, thereby potentially impacting on a larger segment of the population, including those for whom harm occurs below the clinical threshold of pathology [9,10,11,12]. Low-risk gambling limits can inform the development of quantitative guidelines that can usefully augment currently available behavioural responsible gambling guidelines [11,15]. Guidelines may generate public discussion about gambling norms, provide the opportunity for consumers to make informed choices about personal risk, and inform the ethical provision of gambling products [11,19]. They can serve as an easy and cost-effective method to screen for people at high-risk for gambling-related harm [16,21,22] by allowing gamblers to compare their current consumption with the guideline [11,19], and generating inferences about the presence of problems [16,21,22,53]. Limits can assist gamblers in reducing their gambling consumption by increasing awareness of what defines risk behaviour, highlighting potential negative consequences of exceeding the limits, and enhancing motivation to employ self-directed change strategies or seek help [11,22]. Low-risk gambling limits can also be employed in population-level surveillance research to monitor the prevalence of gambling-related harm [12,22,24], used to investigate the efficacy of secondary intervention efforts [22], and applied in tertiary intervention settings for gamblers selecting a moderation goal [11,20,21]. Despite these advantages, it is important to note that there have been some concerns about the promotion of low-risk limits in relation to drinking, including the degree of risk tolerated, that consumers may “drink up” to the limit, or that the limits are perceived as a “safe” baseline from which to range upwards in setting personal limits [13,54].

## 5. Conclusions

This is the first study to attempt to identify and evaluate evidence-based low-risk gambling limits for Australia. A relative risk method identified indicators of gambling consumption levels that reliably differentiate gamblers at lower and higher risk of gambling-related harm. This research identified similar limits across population-representative datasets produced by independent research teams from the two most socio-demographically disparate Australian jurisdictions. These limits are consistent with previous research and are able to predict subsequent gambling-related harm. The two limits related to gambling expenditure (gambling expenditure and gambling expenditure as a proportion of income) were consistently the best-performing. At least from a relative risk perspective, there is little utility in increasing these limits; in fact, gambling at any level appears to carry some level of risk for harm. These limits can serve as working guidelines for the consideration of researchers, clinicians, and policy makers but need to be subject to further rigorous empirical investigation.

## Figures and Tables

**Table 1:** Methodological details for the existing datasets employed in this study.

	Tasmanian SEIS Surveys (%)		Survey on Gambling, Health and Wellbeing in the ACT (%)	Tasmanian Longitudinal Gambling Study		
	Second SEIS	Third SEIS		Wave 1 (%)	Wave 2 (%)	Wave 3 (%)
Sample size	4303	5000	2294	2027	1039	820
Dates of data collection	7 February–3 March 2011	16 September–27 October 2013	18 November–11 February 2015	7 February–3 March 2011	6 November–22 December 2013	19 November–21 December 2014
Sampling	Disproportionate stratified sample design (selected LGAs of high EGM density and high and low SES over-sampled)		Random digit dialling of landline telephone numbers, including listed and unlisted numbers; subsample (32% of total sample) selected based on past-year gambling frequency and overall net gambling expenditure	Sub-sample (47% of total sample) administered a supplementary survey in the 2nd SEIS; comprised all gamblers with PGSI score >0 and past-year EGM gamblers and a randomly selected one-third of non-gamblers and non-problem gamblers	In-scope sample was Wave 1 respondents who agreed to be re-contacted (*n* = 1879)	In-scope sample was Wave 2 respondents who agreed to be recontacted, and those who were unable to be interviewed in Wave 2 but remained a valid contact (*n* = 1269)
	Random digit dialling and exchange-based telephone survey of registered landline telephone numbers	Semi-random dual-frame design (3500 randomly generated landline sample; 1500 non-random list-based mobile telephone sample)				
Interview length (mins)	15.8	15.0	Not reported		24.2	26.2
Participation rate	48.8% ^a^	54.6% ^a^	Not reported		82.1% ^b^	84.4% ^b^
Weighting	A two-stage weighting approach: (a) post-stratification weight using raking approach to adjust for the disproportionate nature of the sample and differential survey response rates across age, gender, educational attainment, country of birth, geographical location and telephone status using independent population benchmarks, and (b) design weight for each frame that included typical adjustments relating to the number of in-scope people in each household and number of landline telephone connections per household; An additional pre-weight calculated to adjust for overlapping chances of selection for persons with both landline and mobile telephones into both sample frames by adjusting for telephone status of sample members to population parameters		Analyses for the full sample (*n* = 7068) were weighted to reflect the age, gender, and marital status of the adult ACT population; analyses for the sub-sample also addressed the oversampling of non-gamblers, high frequency gamblers, and high expenditure gamblers	Weights were generated for the Waves 2 and 3 survey data using raking procedures using benchmarks based on Wave 1		
Ethics approval numbers	University of Melbourne Human Research Ethics Committee (1135477.1/1135477.2)	University of Melbourne Human Research Ethics Committee (1340411)	Australian National University Human Research Ethics Committee (2014/580)	University of Melbourne Human Research Ethics Committee (1340411)		
Sample description	Male (56.4%); Age: 18–34 (10.7%), 35–49 (21.6%), 50–64 (34.7%), 65+ (32.0%); PGSI category ^c^: NG (35.9%), NPG (57.2%), LRG (4.2%), MRG (1.8%), PG (0.5%)		Male (47.0%); Age: 18–34 (13.7%), 35–49 (22.4%), 50–64 (28.4%), 65+ (32.2%); PGSI category ^c^: NG (46.6%), NPG (44.5%), LRG (5.7%), MRG (2.1%), PG (1.1%)	Male (63.1%); Age: 18–34 (7.5%), 35–49 (21.2%), 50–64 (38.3%), 65+ (32.3%); PGSI category ^c^: NG (24.4%), NPG (58.5%), LRG (10.3%), MRG (4.9%), PG (1.6%)	Male (63.1%); Age: 18–34 (6.3%), 35–49 (17.9%), 50–64 (35.2%), 65+ (40.7%); PGSI category ^c^: NG (31.6%), NPG (59.2%), LRG (6.7%), MRG (2.1%), PG (0.4%)	Male (63.1%); Age: 18–34 (6.3%), 35–49 (15.9%), 50–64 (34.8%), 65+ (43.1%); PGSI category ^c^: NG (29.3%), NPG (60.5%), LRG (5.6%), MRG (3.5%), PG (0.7%)

^a^ Overall survey participation rate defined as the number of completed interviews divided by the sum of the completed interviews plus refusals; ^b^ Consent rate defined as the number of completed interviews as a percentage of the number of in-scope people actually contacted; ^c^ PGSI (Problem Gambling Severity Index) category: NG (non-gambling), NPG (non-problem gambling), LRG (low-risk gambling), MRG (moderate-risk gambling), PG (problem gambling).

**Table 2:** Receiver operating characteristic (ROC) analyses for the Tasmanian and ACT data ^a,b^.

Low-Risk Gambling Limit		Tasmanian Dataset	ACT Dataset
Proportion of population (including non-gamblers) reporting gambling-related harm		2.30% (95% CI 1.81, 2.91)	1.92% (95% CI 1.39, 2.65)
Proportion of gamblers reporting gambling-related harm		3.68% (95% CI 2.90, 4.64)	3.54% (95% CI 2.56, 4.88)
Gambling frequency per year	Cut off	30	20
	AUC (95% CI)	0.76 (0.70, 0.81)	0.79 (0.72, 0.86)
	sensitivity, specificity	0.71, 0.67	0.75, 0.69
	N	5754	1215
	%of population exceeding limit ^c^	24.4	18.8
	%of gamblers exceeding limit	39.2	34.7
Gambling expenditure per year	Cut off	615	380
	AUC (95% CI)	0.86 (0.82, 0.90)	0.84 (0.78, 0.91)
	sensitivity, specificity	0.78, 0.77	0.78, 0.74
	N	5498	1157
	%of population exceeding limit ^c^	15.8	14.8
	%of gamblers exceeding limit	25.8	27.9
Gambling expenditure as proportion of gross personal income	Cut off	1.68	0.83
	AUC (95% CI)	0.84 (0.79, 0.89)	0.85 (0.77, 0.92)
	sensitivity, specificity	0.78, 0.74	0.77, 0.76
	N	4954	1014
	%of population exceeding limit ^c^	16.3	13.7
	%of gamblers exceeding limit	17.7	27.4
Number of types of gambling activities	Cut off	2	2
	AUC (95% CI)	0.78 (0.73, 0.83)	0.73 (0.65, 0.82)
	sensitivity, specificity	0.82, 0.58	0.69, 0.65
	N	5860	1208
	%of population exceeding limit	37.3	26.6
	%of gamblers exceeding limit	59.7	49.2

^a^ Endorsement of two or more the Problem Gambling Severity Index (PGSI) negative consequence items selected as definition of harm for derivation of low-risk gambling limits; ^b^ ROC = Receiver Operating Curve; Cut-off = low-risk gambling limit identified by the ROC analysis; AUC = Area Under Curve; 95% CI = 95% Confidence Interval; ^c^ Entire sample in dataset (including non-gamblers).

**Table 3:** Longitudinal prediction of gambling-related harm by the low-risk gambling limits in the Tasmanian Longitudinal Gambling Study ^a,b^.

Low-Risk Gambling Limit		OR	95% CI	*p*	r^2^	OR	95% CI	*p*	r^2^
		Adjusted for Socio-Demographics ^c^				Adjusted for Other Limits/ Socio-Demographic ^d,e^			
		Wave 1 to Wave 2							
Gambling frequency per year	30 times per year	6.11	(1.38, 26.95)	0.017	0.07	1.91	(0.51, 7.12)	0.335	0.14
Gambling expenditure per year	AUD $615 per year	14.23	(2.34, 86.43)	0.004	0.13	10.67	(3.41, 33.36)	<0.001	
Gambling expenditure as proportion of gross personal income	1.68% of gross personal income	15.39	(2.57, 92.23)	0.003	0.17				
Number of types of gambling episode	2 types of gambling activities	2.82	(0.51, 15.55)	0.234	0.03	0.83	(0.11, 6.07)	0.854	
		Wave 1 to Wave 3							
Gambling frequency per year	30 times per year	14.26	(3.24, 62.63)	<0.001	0.18	20.45	(2.68, 156.2)	0.004	0.21
Gambling expenditure per year	AUD $615 per year	3.54	(0.57, 22.13)	0.177	0.12	1.32	(0.13, 13.39)	0.812	
Gambling expenditure as proportion of gross personal income	1.68% of gross personal income	21.11	(4.56, 97.86)	<0.001	0.25				
Number of types of gambling episode	2 types of gambling activities	1.22	(0.37, 4.02)	0.748	0.08	0.6	(0.12, 3.03)	0.534	

^a^ Endorsement of two or more the Problem Gambling Severity Index (PGSI) negative consequence items selected as definition of harm for derivation of low-risk gambling limits; ^b^ OR = Odds Ratio; 95% CI = 95% Confidence Interval; AUD = Australian Dollars; ^c^ separate regressions predicting gambling-related harm by each of the low-risk gambling limits after controlling for socio-demographic characteristics (age, gender, education, country of birth); ^d^ prediction of gambling-related harm by each low-risk gambling limit after controlling for the other low-risk gambling limits and socio-demographic characteristics (age gender, education, country of birth); ^e^ Gambling expenditure as a proportion of gross personal income limit removed due to multicollinearity with gambling expenditure limit.

**Table 4:** The relative and absolute risk for gambling-related harm associated with exceeding the low-risk gambling limits ^a^.

	Gambling Frequency Per Year			Gambling Expenditure Per Year			Gambling Expenditure as Proportion of Gross Personal Income			Number of Gambling Activities		
	Limit	RR ^b^	AR ^b^	Limit	RR ^b^	AR ^b^	Limit	RR ^b^	AR ^b^	Limit	RR ^b^	AR ^b^
Tasmanian Data												
0.25 × limit	8	6.25	4.03	154	9.04	4.41	0.42	7.51	4.29			
0.5 × limit	15	3.64	4.40	308	6.52	5.21	0.84	8.09	5.28	1	-	-
0.75 × limit	23	3.39	4.61	462	6.77	6.16	1.26	7.96	6.08			
At limit	30	3.36	4.91	615	6.47	6.98	1.68	7.48	6.77	2	4.49	4.67
1.5 × limit	45	3.04	5.01	923	7.42	8.87	2.52	7.22	7.99	3	3.70	6.40
2 × limit	60	4.10	7.44	1230	9.39	11.34	3.36	9.00	9.79	4	5.33	10.66
3 × limit	90	4.62	9.38	1845	9.46	14.34	5.04	8.21	11.44	6	4.92	14.71
4 × limit	120	5.58	12.42	2460	10.50	17.15	6.72	9.31	13.91			
5 × limit	150	5.95	13.95	3075	11.09	19.50	8.40	9.09	15.42			
6 × limit	180	6.90	16.96	3690	11.28	21.32	10.08	8.94	16.47			
7 × limit	210	7.48	18.78	4305	11.66	22.77	11.76	8.79	17.31			
8 × limit	240	7.75	20.14	4920	12.12	24.24	13.44	8.93	18.53			
9 × limit	270	8.29	22.02	5535	13.20	26.55	15.12	9.13	19.42			
10 × limit	300	7.89	21.59	6150	12.03	25.47	16.80	10.26	21.91			
20 × limit				12,300	14.11	33.80	33.60	12.63	31.25			
30 × limit				18,450	12.25	31.25	50.40	8.87	24.14			
40 × limit				24,600	9.30	25.00						
**ACT Data**												
0.25 × limit	5	9.46	9.46	95	20.09	10.75	0.21	20.96	11.55			
0.5 × limit	10	8.93	10.63	190	12.55	12.42	0.42	33.98	14.10	1	-	-
0.75 × limit	15	9.29	12.13	285	10.82	14.21	0.62	21.35	15.61			
At limit	20	6.38	12.23	380	11.29	15.42	0.83	20.23	17.06	2	3.64	10.65
1.5 × limit	30	5.36	13.10	570	11.85	17.72	1.25	11.43	18.87	3	4.12	15.36
2 × limit	40	4.74	13.20	760	11.76	19.47	1.66	9.14	20.39	4	4.59	22.75
3 × limit	60	5.68	18.13	1140	10.86	22.70	2.49	5.87	21.01	6	5.52	37.50
4 × limit	80	5.76	21.43	1520	9.56	25.83	3.32	5.43	21.95			
5 × limit	100	4.98	20.76	1900	9.58	28.10	4.15	5.36	23.43			
6 × limit	120	4.61	23.65	2280	9.16	29.89	4.98	6.14	27.27			
7 × limit	140	4.51	24.06	2660	10.48	34.19	5.81	5.91	28.81			
8 × limit	160	4.26	24.75	3040	9.16	34.78	6.64	5.63	28.97			
9 × limit	180	4.28	25.93	3420	8.76	35.43	7.47	6.43	32.63			
10 × limit	200	4.42	27.03	3800	8.38	36.61	8.30	6.01	32.18			
20 × limit				7600	8.30	48.84	16.60	8.08	43.48			
30 × limit				11,400	9.96	63.64	24.90	5.62	37.50			

^a^ Endorsement of two or more PGSI negative consequence items selected as definition of harm for derivation of low-risk gambling limits; ^b^ RR: relative risk ratio, AR: absolute risk.

**Table 5:** Positive and negative predictive values based on the prevalence of gambling-related harm in the population and gambling samples ^a,b^.

Low-Risk Gambling Limit	Positive and Negative Predictive Values Based on the Prevalence of Gambling-Related Harm in the Population (Including Non-Gamblers)				Positive and Negative Predictive Values Based on the Prevalence of Gambling-Related Harm in Gamblers			
	Tasmanian Data (Prevalence = 2.30%)		ACT Data (Prevalence = 1.92%)		Tasmanian Data (Prevalence = 3.68%)		ACT Data (Prevalence = 3.54%)	
	PPV (%)	NPV (%)	PPV (%)	NPV (%)	PPV ^c^ (%)	NPV (%)	PPV ^c^ (%)	NPV (%)
Gambling frequency per year	4.82	98.99	4.52	99.30	7.60	98.37	8.15	98.69
Gambling expenditure per year	7.39	99.33	5.55	99.42	11.47	98.92	9.92	98.92
Gambling expenditure as proportion of gross personal income	6.60	99.30	5.91	99.41	10.28	98.88	10.53	98.90
Number of gambling activities	4.39	99.27	3.72	99.08	6.94	98.83	6.75	98.28

^a^ Endorsement of two or more PGSI negative consequence items selected as definition of harm for derivation of low-risk gambling limits; ^b^ PPV: positive predictive values, NPV: negative predictive values; ^c^ These positive predictive values differ from the absolute risk estimates presented previously due to the use of weighting employed in these analyses.

**Table 6:** Receiver Operating Characteristic (ROC) analyses maximizing sensitivity and specificity ^a,b^.

Low-Risk Gambling Limit		Maximising Sensitivity		Maximising Specificity	
		Tasmanian Data	ACT Data	Tasmanian Data	ACT Data
Proportion of population (including non-gamblers) reporting gambling-related harm		2.30% (95% CI 1.81, 2.91)	1.92% (95% CI 1.39, 2.65)	2.30% (95% CI 1.81, 2.91)	1.92% (95% CI 1.39, 2.65)
Proportion of gamblers reporting gambling-related harm		3.68% (95% CI 2.90, 4.64)	3.54% (95% CI 2.56, 4.88)	3.68% (95% CI 2.90, 4.64)	3.54% (95% CI 2.56, 4.88)
Gambling frequency per year	Cut off	16	10	65	49
	AUC (95% CI)	0.76 (0.70, 0.81)	0.79 (0.72, 0.86)	0.76 (0.70, 0.81)	0.79 (0.72, 0.86)
	sensitivity, specificity	0.84, 0.51	0.88, 0.51	0.50, 0.83	0.50, 0.86
	N	5754	1215	5754	1215
Gambling expenditure per year	Cut off	169	115	2306	1380
	AUC (95% CI)	0.86 (0.82, 0.90)	0.84 (0.78, 0.91)	0.86 (0.82, 0.90)	0.84 (0.78, 0.91)
	sensitivity, specificity	0.94, 0.50	0.93, 0.50	0.50, 0.93	0.51, 0.91
	N	5498	1157	5498	1157
Gambling expenditure as proportion of gross personal income	Cut off	0.51	0.24	6.19	3.03
	AUC (95% CI)	0.84 (0.79, 0.89)	0.85 (0.77, 0.92)	0.84 (0.79, 0.89)	0.85 (0.77, 0.92)
	sensitivity, specificity	0.93, 0.50	0.93, 0.50	0.50, 0.91	0.50, 0.92
	N	4954	1014	4954	1014
Number of types of gambling activities	Cut off	2	2	3	2
	AUC (95% CI)	0.78 (0.73, 0.83)	0.73 (0.65, 0.82)	0.78 (0.73, 0.83)	0.73 (0.65, 0.82)
	sensitivity, specificity	0.82, 0.58	0.69, 0.65	0.54, 0.83	0.69, 0.65
	N	5860	1208	5860	1208

^a^ Endorsement of two or more PGSI negative consequence items selected as definition of harm for derivation of low-risk gambling limits; ^b^ ROC = Receiver Operating Curve; Cut-off = low-risk gambling limit identified by the ROC analysis; AUC = Area Under Curve; 95% CI = 95% Confidence Interval.

**Table 7:** Positive and negative predictive values based on the prevalence of gambling-related harm in the population and gambling samples after maximising sensitivity and specificity ^a,b^.

Low-Risk Gambling Limit	Positive and Negative Predictive Values Based on the Prevalence of Gambling-Related Harm in the Population (Including Non-Gamblers)				Positive and Negative Predictive Values Based on the Prevalence of Gambling-Related Harm in Gamblers			
	Tasmanian Data (Prevalence = 2.30%)		ACT Data (Prevalence = 1.92%)		Tasmanian Data (Prevalence = 3.68%)		ACT Data (Prevalence = 3.54%)	
	PPV (%)	NPV (%)	PPV (%)	NPV (%)	PPV (%)	NPV (%)	PPV (%)	NPV (%)
Maximising Sensitivity								
Gambling frequency per year	3.88	99.27	3.40	99.54	6.15	98.82	6.18	99.14
Gambling expenditure per year	4.24	99.72	3.51	99.73	6.70	99.54	6.39	99.49
Gambling expenditure as proportion of gross personal income	4.20	99.67	3.51	99.73	6.63	99.47	6.39	99.49
Number of gambling activities	4.39	99.27	3.72	99.08	6.94	98.83	6.75	98.28
**Maximising Specificity**								
Gambling frequency per year	6.48	98.60	6.53	98.87	10.10	97.75	11.59	97.91
Gambling expenditure per year	14.39	98.75	9.99	98.96	21.44	97.99	17.22	98.06
Gambling expenditure as proportion of gross personal income	11.57	98.72	10.90	98.95	17.51	97.94	18.66	98.04
Number of gambling activities	6.96	98.71	3.72	99.08	10.82	97.93	6.75	98.28

^a^ Endorsement of two or more PGSI negative consequence items selected as definition of harm for derivation of low-risk gambling limits; ^b^ PPV: positive predictive values, NPV: negative predictive values.

## Data Availability

The data presented in this study are available on request from the corresponding author. The data are not publicly available due to privacy and ethical restrictions.

## Supplementary Materials

The following are available online at https://www.mdpi.com/2077-0383/10/2/167/s1, Table S1: Demographic and gambling involvement profiles in Tasmania compared to national estimates. Table S2: Gambling indices employed in this study. Table S3. Classification of PGSI items as negative consequences or behavioural symptoms of gambling. Figure S1. Positive and negative predictive values for each low-risk gambling limit based on gambling-related harm for the Tasmanian and ACT data.
