# Supplementary material for: The Development of Empirically Derived Australian Low-Risk Gambling Limits
Source: J Clin Med. 2021 Jan 6;10(2):167. doi: 10.3390/jcm10020167 (PMC7824838; doi:10.3390/jcm10020167)
Supplement: Supplementary file 1 [file jcm-10-00167-s001.pdf]

Supplementary material

Table S1. Demographic and gambling involvement profiles in Tasmania compared to national estimates.

	**Tasmania** [a,b]	**ACT**[a,b]	**Australia** [a,c]
Proportion of Australian population (%)	2.3	1.7	
Median age	40	34	37
Gender (% males)	49.0	49.5	49.4
Number of children per family	1.8	1.8	1.9
Not married (%)	40.4	41.0	41.3
Average people per household	2.4	2.6	2.6
Single person household (%)	28.0	23.4	24.3
Working age (15–64 years) (%)	64.9	70.6	66.7
Elderly population (65+) (%)	16.7	10.7	14.0
Education (secondary school or higher) (%)	21.0	52.9	42.1
Unemployment (%)	6.4	3.6	5.6
Indigenous (%)	4.0	1.4	2.5
Country of birth (Australia) (%)	83.6	71.4	69.8
Australian ancestry (%)	33.9	26.6	25.4
No religion (%)	28.6	28.9	22.3
Median weekly personal income	499	918	577
Median weekly household income	948	1920	1234
Annual gambling participation (%)	61.2	55.1	63.9
Electronic gaming machines (EGMs)	18.6	19.9	20.7
Horse or greyhound	10.5	17.6	15.9
Lotteries	43.0	33.4	49.2
Instant scratch tickets	20.6	15.1	22.0
Keno	26.0	2.9	7.2
Casino table games	6.3	2.8	5.9
Bingo	1.7	2.2	3.0
Sporting or other events	4.4	6.9	5.7
Informal private games	2.6	3.7	3.1
Any PGSI endorsement (%)	6.2	5.4	5.3
Low risk gambling	3.9	3.9	3.0
Moderate risk gambling	1.8	1.1	1.9
Problem gambling	0.5	0.4	0.4

[a] Socio-demographic characteristics [44]; [b] Gambling characteristics [35,37]; [c] Gambling characteristics [6].

Table S2. Gambling indices employed in this study.

Gambling indices[a]	Tasmanian Surveys	ACT Survey
Gambling frequency	Typical wording was: 'In the last 12 months, how many times per week, per month or per year have you played/bet on [gambling activity]?' for different modalities (e.g., venue, telephone, racetrack, off-course venue, internet) of each gambling activity. Annual gambling frequency was calculated by standardising each response to an estimated yearly frequency then summing these yearly frequencies across gambling activities.	Typical wording was: 'In the last 12 months, how many times per week or per month or per year have you played [gambling activity]?' for different modalities (e.g., venue, telephone, racetrack, off-course venue, internet) of each gambling activity. Annual gambling frequency was calculated by standardising each response to an estimated yearly frequency then summing these yearly frequencies across gambling activities.
Gambling expenditure	Typical wording was: 'In the past 12 months, approximately how much money, on average, did you spend during each session of [gambling activity]? For each specific gambling activity'. Total gambling expenditure was calculated by multiplying the gambling frequency with session expenditure estimates for each activity then summing these yearly gambling expenditures across all gambling activities. Gambling expenditure was assessed only in terms of amount of money lost.	Typical wording was: 'Subtracting any winnings, how much money did you spend on [gambling activity] in an average week/an average month in the last 12 months?' for different modalities (e.g., venue, telephone, racetrack, off-course venue, internet). Total annual gambling expenditure was calculated by standardising each response to an estimated yearly expenditure then summing these yearly expenditures across gambling activities. Winnings were recorded; but these were recoded to zero to indicate no gambling expenditure.
Gambling expenditure as a proportion of gross annual personal income	Gross annual personal income was assessed: 'Could you please tell me your approximate annual personal income before tax?' Response options were: Less than $25,000; $25,000–$39,999; $40,000–$64,999; $65,000–$79,999; $80,000–$129,999; $130,000 or more. To derive expenditure as a proportion of income, we used the mid-point of each category's range to represent the respective income category (e.g. $25,000 to $39,999 became $32,500). For the final income category in which no mid-point exists, the same interval that was applied to the preceding category was applied. Total annual gambling expenditure as the divided by the mid-point income value to derive gambling expenditure as a proportion of	Gross annual personal income was assessed: 'Which of the following categories contains your total annual personal income from all sources before tax?' Response options were: negative or zero income; $1–$9,999; $10,000–$19,999; $20,000–$29,999; $30,000–$39,000; $40,000–$49,999; $50,000–$59,999; $60,000–$79,999; $80,000–$99,999; $100,000–$124,999; $125,000–$149,999; $150,000–$199,999; $200,000 or more. To derive expenditure as a proportion of income, we used the mid-point of each category's range to represent the respective income category (e.g. $20,000 to $29,999 became $25,000). For the final income category in which no mid-point exists, the same interval that was applied to the preceding category was applied. Total gambling

	income. A small number of estimates exceeding 100% (n = 17) were removed from the dataset.	expenditure was then divided by the mid-point income value to derive gambling expenditure as a proportion of income. A small number of derived expenditure as a proportion of income estimates exceeding 100% (n = 2) were removed from the dataset.
Number of types of gambling activities	The number of gambling activities was based on participation across each gambling activity, with the exception of informal private games due to low participation. Typical wording was: 'I am going to start by reading a list of popular gambling activities and find out if you have played them for money in the previous 12 months. In the last 12 months, have you…?'	The number of gambling activities were based on participation across each gambling activity, with the exception of informal private games due to low participation. Typical wording was: 'I'm going to read out a list of popular gambling activities that people can do in a number of ways, such as gambling at a venue, by phone, and over the internet. Could you please tell me which of these you have participated in FOR MONEY, during the last 12 months?'

[a] Unless otherwise indicated, all gambling indices were based on estimates from nine gambling activities: EGMs, horse or greyhound racing, instant scratch tickets, lotteries, keno, casino table games, bingo, sports or other event betting, and informal private games.

Table S3. Classification of PGSI items as negative consequences or behavioural symptoms of gambling.

Item	Item classification	
	**Currie, Miller et al. [12]**	**Ferris and Wynne [3]**
1. Have you bet more than you could really afford to lose?	Negative consequence	Behavioural symptom
2. Have you needed to gamble with larger amounts of money to get the same feeling of excitement?	Behavioural symptom	Behavioural symptom
3. When you gambled, did you go back another day to try to win back the money you lost?	Behavioural symptom	Behavioural symptom
4. Have you borrowed money or sold anything to get money to gamble?	Negative consequence	Behavioural symptom
5. Have you felt that you might have a problem with gambling?	Negative consequence	Behavioural symptom
6. Has gambling caused you any health problems, including stress or anxiety?	Negative consequence	Negative consequence
7. Have people criticised your betting or told you that you had a gambling problem, regardless of whether or not you thought it was true?	Negative consequence	Negative consequence
8. Has your gambling caused any financial problems for you or your household?	Negative consequence	Negative consequence
9. Have you felt guilty about the way you gamble or what happens when you gamble?	Negative consequence	Negative consequence

Note: The questions are asked concerning the past 12 months, with answers: Never (0 points), Some of the time (1 point), Most of the time (2 points), Almost always (3 points).

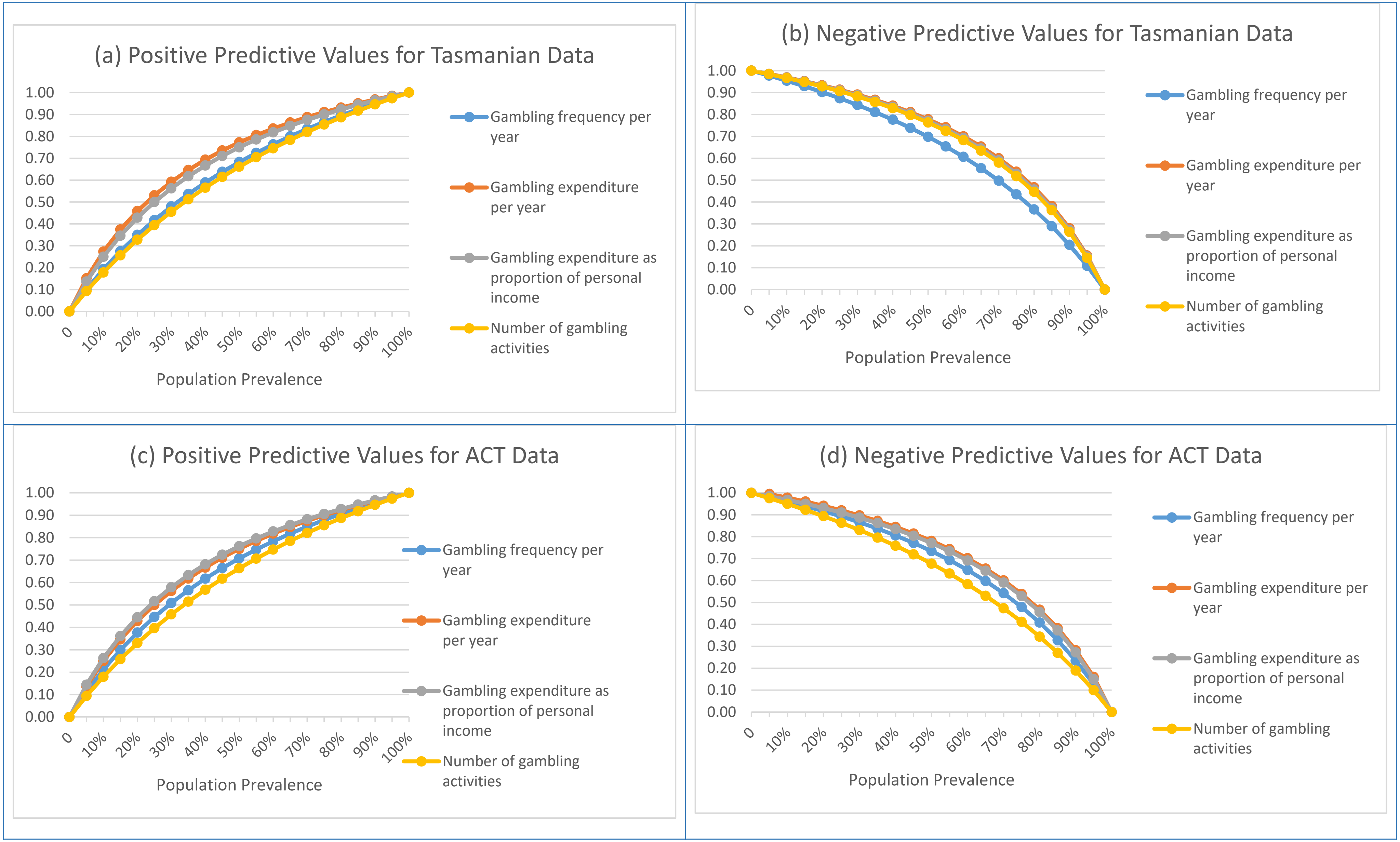
(a) Positive Predictive Values for Tasmanian Data
1.00
0.90
0.80
0.70
0.60
0.50
0.40
0.30
0.20
0.10
0.00
0
10%
20%
30%
40%
50%
60%
70%
80%
90%
100%
Population Prevalence
Gambling frequency per year
Gambling expenditure per year
Gambling expenditure as proportion of personal income
Number of gambling activities
(b) Negative Predictive Values for Tasmanian Data
Population Prevalence
Gambling frequency per year
Gambling expenditure per year
Gambling expenditure as proportion of personal income
Number of gambling activities
(c) Positive Predictive Values for ACT Data
Population Prevalence
Gambling frequency per year
Gambling expenditure per year
Gambling expenditure as proportion of personal income
Number of gambling activities
(d) Negative Predictive Values for ACT Data
Population Prevalence
Gambling frequency per year
Gambling expenditure per year
Gambling expenditure as proportion of personal income
Number of gambling activities

Figure S1. Positive and negative predictive values for each low-risk gambling limit based on gambling-related harm for the Tasmanian and ACT data: (a) Positive Predictive Values for Tasmanian Data; (b) Negative Predictive Values for Tasmanian Data; (c) Positive Predictive Values for ACT Data; (d) Negative Predictive Values for ACT Data.
